# B-1a cells mitigate radiation injury by protecting intestinal barrier integrity

**DOI:** 10.3389/fimmu.2026.1761007

**Published:** 2026-02-04

**Authors:** Tomoki Abe, Atsushi Murao, Satoshi Yamaga, Ping Wang, Monowar Aziz

**Affiliations:** 1 Center for Immunology and Inflammation, The Feinstein Institutes for Medical Research, Manhasset, NY, United States; 2 Departments of Surgery and Molecular Medicine, Zucker School of Medicine at Hofstra/Northwell, Manhasset, NY, United States

**Keywords:** B-1a cells, inflammation, intestine, radiation injury, TGF, tight junction protein

## Abstract

**Introduction:**

Ionizing radiation causes severe gastrointestinal injury. B-1a cells, predominantly located in the peritoneal cavity (PerC), play a critical role in maintaining tissue homeostasis through the secretion of cytokines and natural antibodies. We aim to investigate the status of B-1a cells after irradiation, and their role in ameliorating radiation-induced intestinal injury.

**Methods:**

C57BL/6 mice were exposed to 12-Gy partial body irradiation (PBI) and B-1a cells in the PerC, spleen and bone marrow were determined by flow cytometry. After 24 hours of PBI, 5×10^5^ B-1a cells were intraperitoneally administered in additional animals. Gut histology, intestinal barrier function, tissue injury markers, and TGF-β levels in PerC and gut tissue were assessed.

**Results:**

Irradiation induced apoptosis in B-1a cells, resulting in depletion of B-1a cell numbers. Irradiation increased apoptotic cells in the crypts, decreased tight junction protein expression, and enhanced intestinal permeability. Adoptive transfer of B-1a cells significantly ameliorated these changes. The number of TGF-β-positive B-1a cells in PerC increased after B-1a cell transfer, accompanied by elevated TGF-β levels in both PerC and gut tissue.

**Conclusion:**

We demonstrated that B-1a cell numbers are significantly decreased following PBI and that B-1a cell treatment alleviates radiation-induced intestinal injury possibly via the increase in TGF-β production.

## Introduction

Ionizing radiation can cause severe health hazards following large-scale exposure events such as nuclear accidents, environmental radiation contamination or nuclear terrorism. High-dose irradiation affects multiple organs, including hematopoietic system and gastrointestinal system (1, 2). Conversely, radiation is a critical therapeutic modality for controlling local tumors and improving survival in cancer patients (3). However, it is also associated with adverse effects on normal tissues, particularly in the gastrointestinal tract, which can be severely affected by abdominal or pelvic irradiation (4). Radiation can directly damage epithelial cells, and these changes can compromise intestinal barrier function, resulting in impaired nutrient absorption and heightened susceptibility to infection (5). Delayed tissue repair after intestinal injury can lead to severe clinical symptoms and systemic complications, making it essential to elucidate the mechanisms of intestinal recovery for both improving the safety of radiotherapy and developing effective interventions following radiation exposure.

The intestinal epithelium is one of the most rapidly renewing tissues in the body, continuously regenerating to maintain barrier integrity and homeostasis (6). This regenerative capacity is primarily driven by Lgr5-positive stem cells located at the base of the crypts, which give rise to all epithelial lineages (7, 8). These regenerative processes are orchestrated by a complex network of signaling pathways and cytokines that coordinate interactions among various cell types within the intestinal microenvironment (9–14). Among these, transforming growth factor-beta (TGF-β) has been identified as a key mediator of tissue repair and inflammation resolution (15). In the small intestine, TGF-β supports epithelial cell survival and regeneration, thereby contributing to the restoration of barrier function after injury (8). TGF-β signaling components are distributed among various intestinal cell types, suggesting that this pathway regulates intestinal homeostasis through coordinated actions of epithelial, mesenchymal, and immune cells (16, 17). Collectively, these findings underscore the pivotal role of TGF-β in maintaining intestinal homeostasis and promoting mucosal repair following damage.

B cells are a crucial component of the immune system, responsible for antibody production and immune memory formation (18). They are broadly classified into two major subsets: B-1 and B-2 cells, which differ in their developmental origins, surface markers (phenotypes), and functions (19). B-2 cells, also known as conventional B cells, are primarily involved in adaptive immune responses. B-1 cells are further divided into B-1a and B-1b subsets based on the expression of CD5, with B-1a cells defined as CD19^+^B220^lo/-^CD23^-^CD5^+^ (20, 21). B-1a cells are a major subset of innate-like lymphocytes predominantly found in the pleural and peritoneal cavities (PerC) (22–26). They play a critical role in maintaining local immune homeostasis through the production of natural antibodies, primarily IgM, and the secretion of anti-inflammatory cytokines (27). We and others have shown that B-1a cells contribute not only to early defense against pathogens such as bacteria and viruses but also to the regulation of inflammatory responses and the maintenance of tissue homeostasis (20–23, 28–31). Additionally, B-1a cells have been reported to express TGF-β, however, this has been observed primarily under limited conditions, such as parasitic infections (32). To date, the role of B-1a cells in radiation-induced intestinal injury remains largely unexplored, and it is unclear how these cells are affected by irradiation and whether they contribute to epithelial repair and intestinal homeostasis. Addressing this knowledge gap is critical for developing novel therapeutic strategies against radiation-induced intestinal injury.

In this study, we investigated the potential of adoptively transferred B-1a cells to ameliorate radiation-induced intestinal injury through TGF-β–mediated mechanisms. Using a partial body irradiation (PBI) mouse model, we examined the effects of B-1a cell transfer on intestinal recovery and the levels of TGF-β in PerC and small intestine tissue. By elucidating the potential protective effects of B-1a cells against radiation-induced intestinal injury, this study aims to advance our understanding of post-irradiation intestinal recovery.

## Materials and methods

### Sex as a biological variable

We did not analyze sex as a biological factor in this study. This research predominantly centered on male mice. Therefore, additional studies are required to explore the potential sex-specific variations.

### Experimental animals

Male C57BL/6 wild-type mice (8-10-week-old) weighing 21–28 g were purchased from the Jackson Laboratory (Bar Harbor, ME). All mice were maintained under specific pathogen-free conditions in a temperature-controlled room with 12-h light/dark cycle and fed a standard animal diet with water. The study was performed following the National Institutes of Health guidelines for the care and use of laboratory animals and approved by Institutional Animal Care and Use Committee of the Feinstein Institutes for Medical Research (Protocol Approval Number: 24-1093).

### Murine model of partial body irradiation

Mice were exposed to a single dose of 12-Gy PBI at a dose rate of ∼1 Gy/min (320 kV, 12.5 mA, 50 cm source-to-skin distance) using an X-ray irradiation system, X-Rad320 (Precision X-Ray Inc., Madison, CT). The PBI dose was determined based on our previous study (33). During irradiation, mice were briefly restrained and placed in a fitted container with the hind extremities (fibula, tibia and feet) shielded using lead tubes, thereby protecting approximately 5% of the bone marrow.

### Adoptive transfer of B-1a cells

Mice were euthanized with CO_2_ asphyxiation, and PerC lavage was collected as described earlier (34). PerC lavage was centrifuged at 400 × *g* for 8 min at 4°C and resuspended in PBS with 0.5% bovine serum albumin and 2 mM EDTA. B-1a cells were isolated from PerC lavage using B-1a Cell Isolation Kit, mouse (Cat. No. 130-097-413, Miltenyi Biotec, Auburn, CA) in accordance with the manufacturer’s recommendations. A total of 5 × 10^5^ B-1a cells in 150 μL of PBS were transferred intraperitoneally one day after irradiation. As vehicle negative control, 150 μL of PBS was injected intraperitoneally. Mice were randomly assigned into three groups: sham, vehicle-treated PBI, and B-1a cell-treated PBI groups. Mice in vehicle- and B-1a cell-treated PBI groups received 12-Gy PBI on day-0. Mice in B-1a cell-treated PBI group were administered an intraperitoneal injection of 5 × 10^5^ B-1a cells on day-1, while vehicle group mice received PBS. Plasma and gut tissues were harvested on day-5. For the measurement of TGF-β levels, PerC lavage was collected on day-3.

### Harvesting cells from the peritoneal cavity, spleen, and bone marrow

Isolation of cells from PerC, spleen, and bone marrow was described previously (34–36). In brief, PerC was instilled with 8–10 mL of PBS supplemented with 2% heat-inactivated FBS. The abdomen was gently agitated, and PerC lavage was aspirated. This process was repeated for a second time. PerC lavage samples were centrifuged at 400 × g for 8 min at 4°C and re-suspended in PBS supplemented with 2% heat-inactivated FBS. The femurs, tibias and spleens were dissected. The bone marrow from the femurs and tibias was flushed with PBS supplemented with 2% heat-inactivated FBS, and both bone marrow and spleen were filtered through a 70-μm cell strainer. Red blood cells were lysed using RBC lysis buffer (Invitrogen, Carlsbad, CA). Suspension of cells was centrifuged at 400 × g for 5 min at 4°C and re-suspended in PBS supplemented with 2% heat-inactivated FBS.

### Flow cytometry

A total of 1 × 10^6^ cells were suspended in 200 μL of PBS supplemented with 2% heat-inactivated FBS. For surface staining, cell suspensions were incubated for 30 min at 4°C with combinations of monoclonal fluorescently conjugated antibodies: PerCP-Cy5.5 anti-mouse CD45 antibody (I3/2.3, Cat. No. 147706, BioLegend, San Diego, CA), APC-Fire750 anti-mouse CD19 antibody (6D5, Cat. No. 115558, BioLegend), APC anti-mouse B220 antibody (RA3-6B2, Cat. No. 103212, BioLegend), FITC anti-mouse B220 antibody (RA3-6B2, Cat. 103205 BioLegend), PE-Cy7 anti-mouse CD23 antibody (B3B4, Cat. No. 101614, BioLegend) and PE anti-mouse CD5 antibody (53-7.3, Cat. No. 100608, BioLegend) for 30 min at 4°C. TruStain FcX™ PLUS (anti-mouse CD16/32) Antibody (S17011E, Cat. No. 156604, BioLegend) was used to prevent nonspecific antibody binding, and the cell viability was determined using a Zombie Aqua Fixable Viability Kit (Cat. No. 423102, BioLegend). After that, B-1a cells (CD45^+^CD19^+^B220^lo/-^CD23^-^CD5^+^) were detected by flow cytometry. For TGF-β intracellular staining, cells are first stimulated with Cell Activation Cocktail (Cat. No. 423303, BioLegend) for 4.5 h at 37°C. After stimulation, the cell underwent surface staining as described above, followed by fixation and permeabilization by using IC Fixation Buffer (Cat. No. 00-8222-49, Invitrogen) and Foxp3/Transcription Factor Staining Buffer Set (Cat. No. 00-5523-00, Invitrogen) according to the manufacture’s protocol. Intracellular staining for TGF-β was then performed using BV421 anti-mouse LAP (TGF-β1) antibody (TW7-16B4, Cat. No. 141408, BioLegend). Unstained cells were used to establish control voltage settings, and single-color compensation was established with BD™ CompBeads Anti-Mouse Ig, κ/Negative Control Compensation Particles Set (Clone 187.1, Cat. No. 552843, BD Biosciences, San Jose, CA). Absolute numbers of cells were determined by adding Precision Count Beads™ (Cat. No. 424902, BioLegend) to the samples according to the manufacturer’s instructions. Acquisition was performed using BD FACSymphony (BD Biosciences) and data were analyzed with FlowJo software v10.10.0 (Tree Star, Ashland, OR).

### *In vitro* assessment of radiation-induced B-1a cell death

As described above, cells were collected from PerC, bone marrow, and spleen of healthy mice. A total of 1 × 10^6^ cells from the entire population were suspended in 200 μL of RPMI supplemented with 10% heat-inactivated FBS and 1% penicillin-streptomycin. The cells were seeded in 96-well plates and exposed to 12-Gy ionizing radiation. After incubation at 37°C with 5% carbon dioxide for 24 h, the cells were surface-stained and subsequently stained for apoptosis using the FITC Annexin V Apoptosis Detection Kit I (Cat. No. 556547, BD Biosciences) according to the manufacturer’s instructions, and analyzed by flow cytometry.

### Gut tissue collection

The entire gut from the stomach to the rectum was harvested. The distal 5 cm from the pyloric end of the stomach was defined as duodenum and discarded. The segment extending 5 cm distal to pylorus to cecum was defined as small intestine, and the region from cecum to rectum was defined as colon. For histological analysis, the proximal 5 cm segment of small intestine was used. For protein analysis and other biochemical assays, an additional 5 cm segment distal to the histological samples was collected.

### Isolation of lymphocytes from intestinal epithelium, lamina propria, and Peyer’s patch

The small intestines were collected for the flow cytometric analysis of lymphocytes from epithelium, lamina propria, and Peyer’s patch. The small intestines were opened immediately after the removal of fat tissues and Peyer’s patches. The contents were gently washed out with cold PBS. Small intestine tissues were cut into small pieces and incubated in RPMI 1640 medium supplemented with 2% FBS, 5 mM EDTA (Cat. No. AM9260G, Thermo Fisher Scientific), and 1 mM DTT (Cat. No. P2325, Thermo Fisher Scientific) for 20 min at room temperature with gentle agitation to isolate the epithelial layer. After samples were filtered through a 100-μm mesh, density gradient centrifugation was performed. The cells suspended in 40% Percoll were layered onto 75% Percoll, and samples were centrifuged at 800 × g for 20 min at 15°C. The interphase layer was collected to obtain intraepithelial lymphocytes. Small intestine tissues remained on the filter were collected to isolate lamina propria lymphocytes. Those tissues were incubated in RPMI 1640 medium supplemented with 2% FBS and 1 mg/mL Collagenase type I (Cat No. LS004194, Worthington Biochemical Corporation, Lakewood, NJ) for 30 min at 37°C with gentle agitation to isolate the lamina propria layer. After samples were filtered through a 100-μm mesh, density gradient centrifugation was performed. The cells suspended in 40% Percoll were layered onto 75% Percoll, and samples were centrifuged at 800 × g for 20 min at 15°C. The interphase layer was collected to obtain lamina propria lymphocytes. Peyer’s patches were filtered through a 70-μm cell strainer. Suspension of cells was centrifuged at 400 × g for 5 min at 4°C and collected as lymphocytes in Peyer’s patch.

### Determination of tissue injury markers

Plasma levels of aspartate aminotransferase (AST) (Cat. No. 23-666-121, Pointe Scientific, Canton, MI), lactate dehydrogenase (LDH) (Cat. No. 23-666-353, Pointe Scientific), and lactate (Cat. No. L7596-50, Pointe Scientific) were determined using specific colorimetric enzymatic assays according to the manufacturer’s instructions. The absorbance was measured using Synergy Neo2 (Agilent Technologies, Santa Clara, CA).

### Enzyme-linked immunosorbent assay

The concentrations of TGF-β in PerC lavage and small intestine tissue were measured using the mouse TGF-β1 DuoSet ELISA kit (Cat. No. DY1697, R&D Systems, Minneapolis, MN) according to manufacturer’s instructions. Small intestine tissues were homogenized in ice-cold lysis buffer containing protease inhibitors, and the supernatants were collected after centrifugation at 13,000 × g for 20 min at 4°C PerC lavage was obtained by washing the peritoneal cavity with 1 ml of PBS supplemented with 2% heat-inactivated FBS, and the supernatants were collected after centrifugation at 450 × g for 8 min at 4°C.

### Fluorescein isothiocyanate-dextran permeability assay

Mice were randomly assigned into three groups: sham, vehicle-treated PBI, and B-1a cell-treated PBI groups. Mice in vehicle- and B-1a cell-treated PBI groups received 12-Gy PBI on day-0. Mice in B-1a cell-treated PBI group received adoptive transfer of B-1a cells on day-1, while vehicle group mice received PBS. On day-5, mice were fasted for 4 h, followed by oral gavage administration of FITC-dextran 4 kDa (FD-4) (Cat. No. 60842-46-8, Sigma-Aldrich, St. Louis, MO) at a dose of 440 mg/kg body weight. After 5 h, whole blood was collected and centrifuged at 10,000 × g for 10 min at 4°C. The resultant plasma (50 μL) was diluted 1:2 with PBS (pH 7.4) and the concentration of FD-4 was measured using fluorescence intensity of FD-4 at an excitation wavelength of 485 nm and an emission wavelength of 528 nm. All samples and standards were run in duplicate.

### Small intestine tissue histology

Formalin fixed and paraffin embedded small intestine blocks were sectioned at 5 µm thickness and placed on glass slides. Small intestine tissue samples were stained with hematoxylin and eosin (H&E). Villi lengths were measured for each sample. Villi length was measured from the tip to base at the crypt-villus junction. For each mouse, 15–20 well-oriented villi were measured using Image J (National Institutes of Health, Bethesda, MD), and the mean value per mouse was calculated. Slides were analyzed using bright field microscopy in a blind manner.

### Terminal deoxynucleotide transferase dUTP nick end labeling assay

To assess apoptotic cells, small intestine tissue sections were stained with *In Situ* Cell Death Detection kit (Cat. No. 11-684-795-910, Roche Diagnostics, Indianapolis, IN) according to the manufacturer’s protocol. Slides were examined by LSM900 (Zeiss, Oberkochen, Germany) microscope, and the average number of TUNEL-positive cells in the crypt was scored.

### Immunofluorescence staining

Paraffin-embedded tissue section (5 µm thickness) was deparaffinized, rehydrated, and subjected to antigen retrieval by heating in citrate buffer for 30 min. After blocking with appropriate serum, sections were incubated for 48 h at 4°C with primary antibodies against ZO-1 (rabbit polyclonal, Cat. No. 40-2200, Invitrogen; dilution 1:30). After washing, sections were incubated for 2 h at room temperature with secondary antibodies Alexa Fluor 647-conjugated anti-rabbit IgG (Cat. No. A-21245, Invitrogen; dilution 1:250). Nuclei were counterstained with NucBlue Live ReadyProbes reagent (Cat. No. R37606, Invitrogen) and were mounted using ProLong Gold Antifade Reagent (Cat. No. P36934, Invitrogen). For quantification of immunofluorescence intensity, three images were captured from each of three independent samples per group using LSM900 (Zeiss) microscope and mean fluorescence intensity was measured by ImageJ (National Institutes of Health).

### Survival assay

The mice were monitored everyday over a 30-day period for survival analysis. The humane criteria established for the present study were as follows: minimal or no response to stimuli, hunched or recumbent posture, grimace score of = 2, body condition score ≤ 2, weight loss of ≥ 20% or bleeding. All animals fulfilling ≥ 2 humane criteria were subjected to euthanasia.

### Statistical analysis

Figure preparation and statistical analysis were performed with GraphPad Prism v10 (GraphPad Software, LLC, San Diego, CA). All data in the figures are presented as the mean ± SEM. Data were compared using one-way analysis of variance (ANOVA) followed by Tukey’s multiple comparison test for multigroup. Survival rates were analyzed by the Kaplan-Meier estimator and compared using a log-rank test. A *p*-value of < 0.05 was considered statistically significant for comparisons between experimental groups.

## Results

### Partial body irradiation decreases B-1a cells in PerC, spleen, and bone marrow

**Figure 1:**
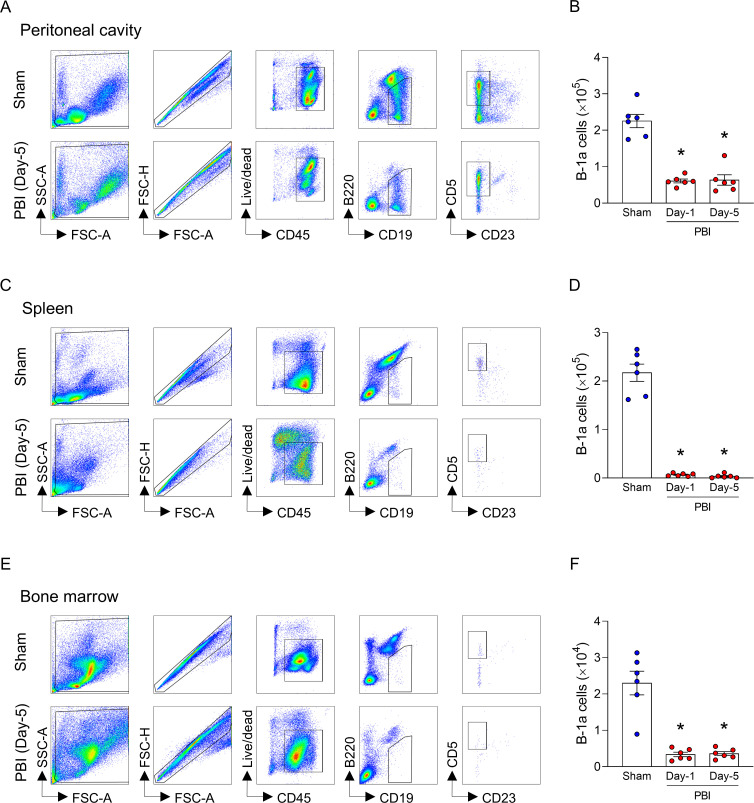
Partial body irradiation reduces the number of B-1a cells in the peritoneal cavity, spleen, and bone marrow. Mice were irradiated with 12-Gy partial body irradiation (PBI) on day-0. Cells from peritoneal cavity (PerC), spleen, and bone marrow were collected on day-1 or day-5. Representative gating strategy of flow cytometry for B-1a cells (CD45^+^CD19^+^B220^lo/-^CD23^-^CD5^+^) from **(A)** PerC, **(C)** spleen, and **(E)** bone marrow. The number of B-1a cells in **(B)** PerC, **(D)** spleen, and **(F)** bone marrow are shown. (n = 6 mice/group). Data are expressed as means ± SEM and were compared using one-way ANOVA followed by Tukey’s multiple comparison test (*p < 0.05 vs. sham). Each experiment was independently performed twice, and all data were used for analysis.

**Figure 2:**
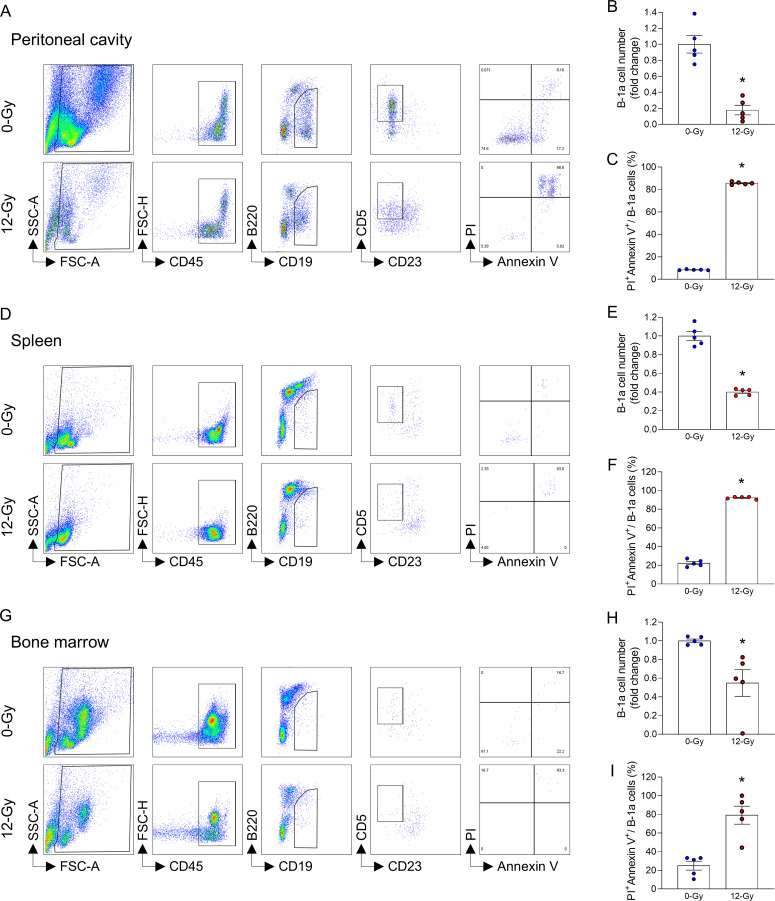
Radiation induces apoptosis and reduces B-1a cell numbers *in vitro*. Cells were collected from peritoneal cavity (PerC), spleen, and bone marrow of healthy mice. A total of 1 × 10^6^ cells were seeded in 96-well plates and exposed to 12-Gy ionizing radiation. After incubation for 24 h, the cells were analyzed by flow cytometry. **(A, D, G)** Representative gating strategy of flow cytometry for B-1a cells (CD45^+^CD19^+^B220^lo/-^CD23^-^CD5^+^) and subsequent PI and Annexin V staining. **(B, E, H)** The number of B-1a cells in **(B)** PerC, **(E)** spleen, and **(H)** bone marrow. **(C, F, I)** Percentages of PI^+^Annexin V^+^ B-1a cells in **(C)** PerC, **(F)** spleen, and **(I)** bone marrow. (n = 5 samples/group). Data are expressed as means ± SEM and were compared using one-way ANOVA followed by Tukey’s multiple comparison test (*p < 0.05 vs. 0-Gy). Each experiment was independently performed twice, and all data were used for analysis.

To investigate the dynamics of B-1a cells after irradiation, the mice were exposed to 12-Gy PBI, and the PerC, spleen, and bone marrow were collected on 1 and 5 days after irradiation. B-1a cells were identified as CD45^+^CD19^+^B220^lo/-^CD23^-^CD5^+^ cells by flow cytometry. In PerC, the number of B-1a cells significantly decreased after PBI (**Figures 1A, B**; **Supplementary Figure 1A**). A similar result was observed in the spleen and bone marrow (**Figures 1C–F**; **Supplementary Figures 1B, C**). These findings indicate that PBI caused a dramatic reduction of B-1a cells in various compartments. To determine the cause of B-1a cell depletion following PBI, in an *in vitro* setting, we exposed the cells collected from PerC, spleen, and bone marrow to 12-Gy ionizing radiation. At 24 hours after radiation exposure, the number of B-1a cells dramatically decreased (**Figures 2A, B, D, E, G, H**). Interestingly, we observed a dramatic increase in B-1a cell apoptosis, as revealed by their co-staining for both PI and Annexin V post-radiation, indicating that B-1a cell depletion occurs due to apoptosis (**Figures 2C, F, I**). Thus, PBI leads to a dramatic reduction in B-1a cell numbers primarily due to apoptosis.

### Adoptive transfer of B-1a cells attenuates radiation-induced intestinal injury

**Figure 3:**
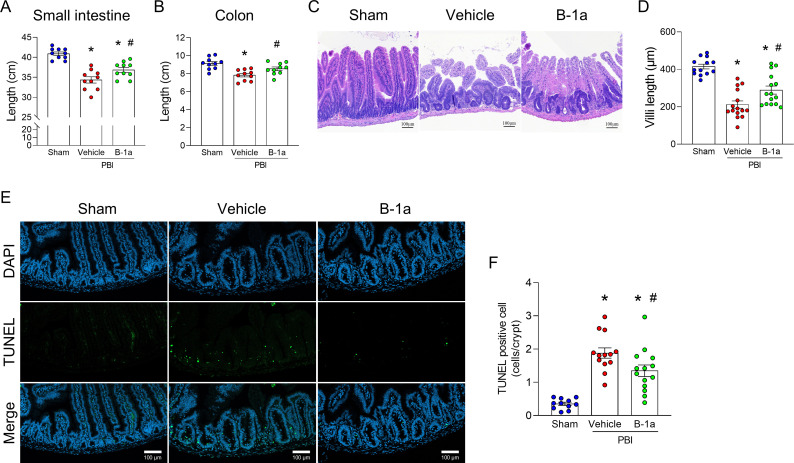
Adoptive transfer of B-1a cells ameliorates radiation-induced intestinal injury. Mice were randomly assigned into three groups: sham, vehicle-treated partial body irradiation (PBI), and B-1a cell-treated PBI groups. Mice in vehicle- and B-1a cell-treated PBI groups received 12-Gy PBI on day-0. Mice in B-1a cell-treated PBI group were administered an intraperitoneal injection of 5 × 10^5^ B-1a cells on day-1, while vehicle group mice received PBS. Gut tissues were harvested on day-5. **(A, B)** The length of **(A)** small intestine and **(B)** colon (n = 10 mice/group). **(C)** Representative images of H&E-stained small intestine. Scale bar: 100 µm. **(D)** Villus length (n = 13–15 mice/group). **(E)** Representative images of TUNEL-stained small intestine. TUNEL (green) and DAPI (blue). Scale bar: 100 µm. **(F)** Number of TUNEL-positive cells per crypt (n = 12–14 mice/group). Data are expressed as means ± SEM and were compared using one-way ANOVA followed by Tukey’s multiple comparison test (*p < 0.05 vs. sham and ^#^*p* < 0.05 vs. vehicle mice). Each experiment was independently performed 3 times, and all data were used for analysis.

We then evaluated whether adoptive transfer of B-1a cells could ameliorate radiation-induced intestinal injury. At 24 hours after exposure of 12-Gy PBI, 5 × 10^5^ B-1a cells isolated from PerC of healthy mice were injected intraperitoneally. On day-5, gut tissues were collected. Both small intestine and colon lengths were shortened in the vehicle-treated PBI mice, whereas this shortening was attenuated in B-1a cell-treated PBI mice (**Figures 3A, B**). Histological analysis of H&E-stained small intestine sections revealed villus shortening in vehicle-treated PBI mice, which was attenuated in B-1a cell-treated PBI mice (**Figures 3C, D**). TUNEL staining revealed an accumulation of apoptotic cells within the crypts of vehicle-treated PBI mice, which was statistically reduced in B-1a cell-treated PBI mice (**Figures 3E, F**). These findings indicate that adoptive transfer of B-1a cells attenuated radiation-induced intestinal tissue injury.

### Adoptive transfer of B-1a cells attenuates radiation-induced intestinal barrier dysfunction, tissue injury, and mortality

**Figure 4:**
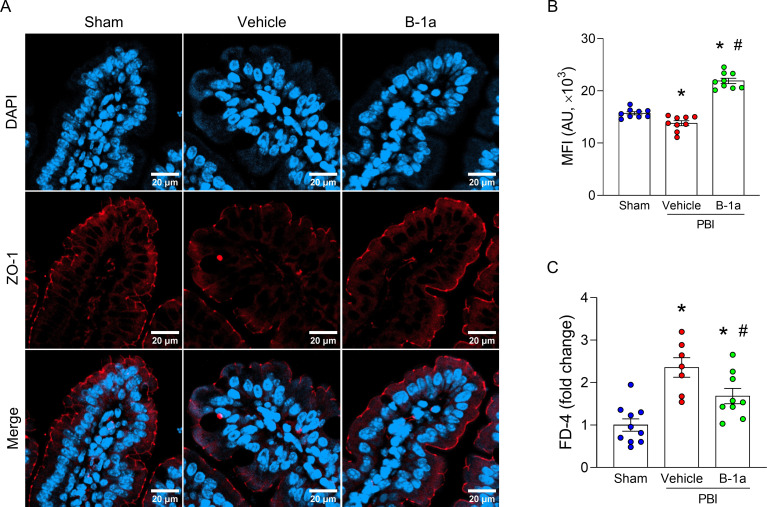
Adoptive transfer of B-1a cells attenuates radiation-induced intestinal barrier dysfunction. Mice were randomly assigned into three groups: sham, vehicle-treated partial body irradiation (PBI), and B-1a cell-treated PBI groups. Mice in vehicle- and B-1a cell-treated PBI groups received 12-Gy PBI on day-0. Mice in B-1a cell-treated PBI group were administered an intraperitoneal injection of 5 × 10^5^ B-1a cells on day-1, while vehicle group mice received PBS. Small intestine tissues and plasma were harvested on day-5. **(A)** Representative immunofluorescence images of ZO-1. Scale bar: 20 µm. **(B)** Mean fluorescence intensity of ZO-1. (n = 9 images/group). **(C)** FITC-dextran intestinal permeability assay. (n = 7–9 mice/group). Data are expressed as means ± SEM and were compared using one-way ANOVA followed by Tukey’s multiple comparison test (*p < 0.05 vs. sham and ^#^*p <* 0.05 vs. vehicle mice). Each experiment was independently performed 3 times, and all data were used for analysis. MFI, mean fluorescent intensity; AU, arbitrary unit; FD-4, fluorescein isothiocyanate-dextran 4 kDa.

**Figure 5:**
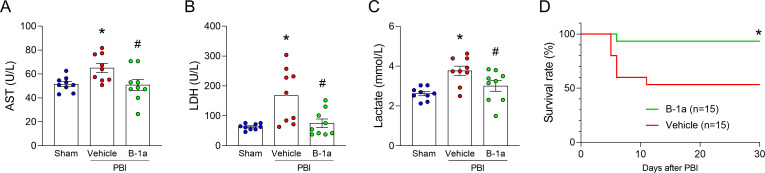
Adoptive transfer of B-1a cells attenuates radiation-induced tissue injury and improves survival. Mice were randomly assigned into three groups: sham, vehicle-treated partial body irradiation (PBI), and B-1a cell-treated PBI groups. Mice in vehicle- and B-1a cell-treated PBI groups received 12-Gy PBI on day 0. Mice in B-1a cell-treated PBI group were administered an intraperitoneal injection of 5 × 10^5^ B-1a cells on day-1, while vehicle group mice received PBS. Plasma was harvested on day 5. **(A-C)** The plasma levels of tissue injury markers. **(A)** Aspartate aminotransferase, **(B)** lactate dehydrogenase, and **(C)** lactate (n = 9 mice/group). Data are expressed as means ± SEM and were compared using one-way ANOVA followed by Tukey’s multiple comparison test (*p < 0.05 vs. sham and ^#^*p <* 0.05 vs. vehicle mice). Each experiment was independently performed 3 times, and all data were used for analysis. **(D)** A 30-day survival study of irradiated mice with or without adoptive transfer of B-1a cells. n = 15 mice/group. Experiments were performed 3 times, and all data were used for analysis. Survival rates were analyzed by the Kaplan-Meier estimator using a log-rank test. (*p < 0.05 vs. vehicle). AST, aspartate aminotransferase; LDH, lactate dehydrogenase.

In immunofluorescence images of small intestinal villi, ZO-1 staining at the apical junction was continuous in sham mice but appeared fragmented and disrupted in vehicle-treated PBI mice. In contrast, the continuity of ZO-1 staining was largely restored in B-1a cell-treated PBI mice (**Figure 4A**). Quantitative analysis of fluorescence intensity further confirmed that ZO-1 levels were significantly higher in B-1a cell-treated PBI mice compared to vehicle-treated PBI mice (**Figure 4B**). Consistent with these findings, FITC-dextran permeability assay showed that radiation-induced intestinal barrier disruption was attenuated by adoptive transfer of B-1a cells (**Figure 4C**). Furthermore, tissue injury markers, including AST, LDH, and lactate, which were elevated in vehicle-treated PBI mice, were reduced in B-1a cell-treated PBI mice (**Figures 5A–C**). In addition, our data clearly showed that the B-1a cell–treated PBI mice had a significantly higher survival rate than the vehicle-treated PBI mice (**Figure 5D**). These findings indicate that adoptive transfer of B-1a cells restored intestinal barrier integrity, thereby reducing tissue injury and enhancing survival following irradiation.

### Adoptive transfer of B-1a cells enhances TGF-β levels in PerC and small intestine

**Figure 6:**
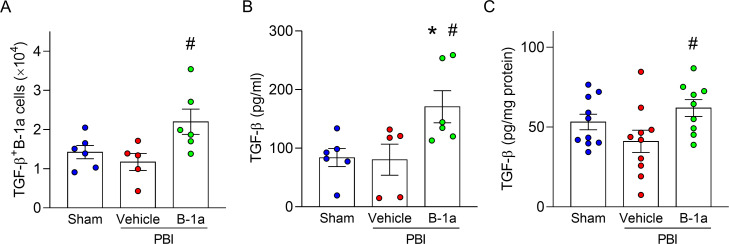
Adoptive transfer of B-1a cells increases TGF-β in peritoneal cavity and small intestine. Mice were randomly assigned into three groups: sham, vehicle-treated partial body irradiation (PBI), and B-1a cell-treated PBI groups. Mice in vehicle- and B-1a cell-treated PBI groups received 12-Gy PBI on day-0. Mice in B-1a cell-treated PBI group were administered an intraperitoneal injection of 5 × 10^5^ B-1a cells on day-1, while vehicle group mice received PBS. Peritoneal cavity (PerC) lavage was collected on day-3, and small intestines were harvested on day-5. **(A)** Flow cytometric analysis of PerC cells. The number of **(A)** TGF-β-positive B-1a cells in PerC. **(B, C)** Concentration of TGF-β in **(B)** PerC and **(C)** small intestine (n = 5–10 mice/group). Data are expressed as means ± SEM and were compared using one-way ANOVA followed by Tukey’s multiple comparison test (*p < 0.05 vs. sham and ^#^*p* < 0.05 vs. vehicle mice). Each experiment was independently performed 3 times, and all data were used for analysis.

We investigated the mechanism by which adoptive transfer of B-1a cells ameliorates radiation-induced intestinal injury. Following 12-Gy PBI exposure and subsequent adoptive transfer of B-1a cells, PerC lavage and small intestine tissues were collected on day-3 and -5, respectively. In the PerC, the number of TGF-β-positive B-1a cells in B-1a cell-treated PBI mice was significantly higher than vehicle-treated PBI mice on day-3 (**Figure 6A**). Next, we measured TGF-β concentrations in supernatant of PerC. TGF-β concentration in PerC was elevated in B-1a cell-treated PBI mice compared with other groups (**Figure 6B**). Furthermore, TGF-β concentration in the small intestine was elevated in the B-1a-treated PBI mice compared to vehicle-treated PBI mice (**Figure 6C**). We collected small intestine and isolated lymphocytes from epithelium, lamina propria, and Peyer’s patch for flow cytometric analysis of B-1 cells. However, B-1a cells were barely detected in any intestinal compartment in all groups (**Supplementary Figures 2A–C**). These results indicate that TGF-β produced by adoptively transferred B-1a cells may contribute to the amelioration of radiation-induced intestinal injury. Notably, the increase in TGF-β concentration in the small intestine appear to result from its release as a soluble factor, rather than from direct infiltration of TGF-β-producing B-1a cells.

## Discussion

**Figure 7:**
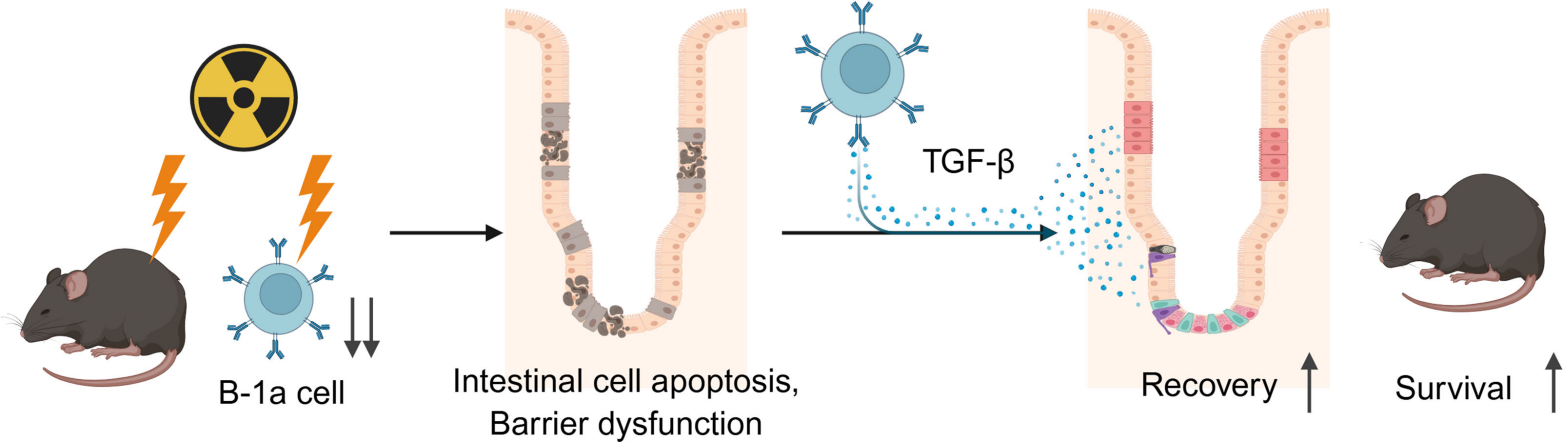
Summary of findings. Partial body irradiation depletes B-1a cells and causes intestinal injury characterized by epithelial cell apoptosis and barrier dysfunction. Adoptive transfer of B-1a cells increases TGF-β levels, which contributes to the suppression of epithelial damage, recovery of intestinal barrier function, and survival improvement.

In the present study, we demonstrate that B-1a cells at various compartments were decreased after ionizing radiation because of their apoptosis. Irradiation shortened villus length, increased apoptotic cells in the crypts, decreased tight junction protein expression, and enhanced intestinal permeability. Adoptive transfer of B-1a cells significantly ameliorated radiation-induced intestinal injury. Furthermore, the number of TGF-β-positive B-1a cells in PerC increased after B-1a cell transfer, accompanied by elevated TGF-β levels in both PerC and gut tissue. TGF-β derived from the transferred B-1a cells may contribute to this beneficial effect (**Figure 7**).

Abdominal irradiation is known to directly damage intestinal epithelial cells through DNA damage and oxidative stress, resulting in cell death, crypt loss, and disruption of barrier function (37–39). In addition to this direct cytotoxic effect, radiation can impair intestinal function indirectly by inducing phenotypic changes and cell death in immune cells, thereby disrupting the homeostasis of the intestinal microenvironment (40, 41). A reduction in regulatory T cells leads to decreased secretion of anti-inflammatory cytokines, such as IL-10 and TGF-β, impairing the resolution of inflammation. Meanwhile, activated M1 macrophages and neutrophils release excessive TNF-α, IL-1β, and reactive oxygen species, exacerbating epithelial cell death and damaging the stem cell niche. In addition, a decrease in innate lymphoid cells type 3 reduces IL-22 production, which is essential for epithelial regeneration, thereby delaying barrier reconstruction. This breakdown of the immune network likely contributes to the chronicity of radiation-induced intestinal injury and sustained barrier dysfunction (42–45). In this study, we showed that irradiation causes apoptosis in B-1a cells. The precise mechanisms underlying radiation-induced immune cell death remain unclear and may involve multiple complex pathways, including ferroptosis, necroptosis and pyroptosis. Further research is warranted to elucidate the mechanisms of radiation-induced immune dysfunction and to explore therapeutic prospects (41, 46).

B-1a cells spontaneously secrete natural IgM, contributing to early defense against pathogens and the clearance of apoptotic cells, and produce anti-inflammatory cytokines such as IL-10 to promote tissue injury resolution. B-1a cells also closely interact with immune cells such as Tregs and macrophages, exerting anti-inflammatory effects both directly and indirectly (27, 47). Our previous studies demonstrated that B-1a cells protect the intestine in ischemic and inflammatory models, partly through IL-10-mediated suppression of excessive inflammation, and partly via GM-CSF and natural IgM supporting immune function and early defense (21–23, 28–31, 34, 48). These factors are well known to play a critical role in maintaining the intestinal epithelium (49, 50). Therefore, the reduction of B-1a cells after irradiation may exacerbate intestinal injury by eliminating a key source of protective signals. In the present study, we observed a marked decrease in peritoneal B-1a cells after irradiation, implicating their loss contributes to impaired epithelial repair and barrier function. Furthermore, adoptive transfer of B-1a cells after irradiation led to improvement of epithelial structure, intestinal function, and tissue injury. These findings indicate that B-1a cells exert multifaceted protective effects against radiation-induced intestinal injury, including barrier maintenance and apoptosis suppression, highlighting their potential as a cellular therapeutic approach. Radiation-induced organ injury such as that affecting the liver and lungs, is well recognized (51, 52). Tissue injury markers, such as AST, LDH, and lactate, correlated well with the severity of histopathological intestinal damage. Their elevated levels in blood indicate that injury to other organs might also have occurred following PBI. In our previous study (28), using an intestinal ischemia model, we observed elevation of these tissue injury markers and histological intestinal injury were accompanied by tissue injury in remote organs, like lungs. These markers were significantly reduced by intraperitoneal adoptive transfer of B-1a cells. Although organs other than the intestine were not directly investigated in the present study, these findings of global tissue injury markers suggest that B-1a cells may also have potential beneficial effects on other organs in the context of radiation-induced injury. On the other hand, although irradiation remarkably reduced PerC B-1a cells, it did not represent complete depletion. Therefore, as we previously reported (21, 30), the use of B-1a cell-deficient CD19 KO mice may provide additional insights into the precise roles and dynamics of B-1a cells under radiation-induced injury.

Moreover, we found that while radiation dramatically decreased total B-1a cell numbers, the reduction in TGF-β-positive B-1a cells was not as pronounced as the overall B-1a cell decrease. This observation could be attributed to two possibilities: either this subset of B-1a cells (i.e., TGF-β-positive B-1a cells) is comparatively resistant to radiation exposure, or that under steady-state conditions (without stimulation), TGF-β expression in B-1a cells is low, but radiation stress induces an increase in TGF-β expression in these cells. Nevertheless, the severe overall depletion of B-1a cells by radiation resulted in a lower absolute number of TGF-β-positive B-1a cells compared to the steady state. In contrast, in the B-1a cell-treated group, the number of TGF-β-positive B-1a cells increased. This increase was likely due to a combination of the B-1a cell transfer itself (increasing the total B-1a cell population) and an elevated TGF-β expression in these cells, stimulated by the stressful microenvironment. This scenario was further supported by elevated TGF-β levels observed in both the peritoneal cavity and intestinal tissues. CD5^+^ B-1a cells are sometimes included in regulatory B (Breg) cells, which are a heterogenous population with diverse phenotype. Breg cells are known to produce IL-10, and have also been shown to produce TGF-β (53). However, only a study has demonstrated TGF-β production by CD5^+^ B-1a cells. Xiao et al. (32) demonstrated *in vitro* that CD5^+^ B-1a cells express TGF-β in the context of immune response to parasites. In this study, we first showed *in vivo* that B-1a cells produce TGF-β, suggesting that B-1a cell-derived TGF-β may contribute to the promotion of epithelial repair and barrier function. TGF-β in the intestine exerts multiple protective effects, including inhibiting apoptosis in intestinal epithelial cells, enhancing tight junction protein expression, promoting Treg induction and anti-inflammatory cytokine production, and regeneration and proliferation of intestinal stem cells, thereby limiting tissue damage and local inflammation after radiation or inflammatory injury (8, 54–60). Previous studies identified macrophages and monocytes as major sources of TGF-β in the intestine (8). Our results reveal that B-1a cells can serve as a novel local source of TGF-β during intestinal regeneration after radiation exposure. Although B-1a cells are abundant in the peritoneal cavity but sparse in intestinal tissues, their adoptive transfer increases local TGF-β levels in the intestinal microenvironment, supporting epithelial repair and barrier maintenance. These findings suggest that multiple immune cell populations collectively provide TGF-β to ensure flexible and efficient intestinal regeneration.

Adoptive transfer of B-1a cells appears to regulate radiation-induced intestinal injury possibly through TGF-β–mediated mechanisms. However, while correlations between increased TGF-β levels and tissue protection were observed, causal relationships remain to be validated. In addition, complex interactions with the local immune environment, including other immune cell populations, may also influence the outcomes. Based on our previous studies in sepsis and intestinal ischemia-reperfusion injury, adoptive transfer of B-1a cells improved disease outcomes by regulating macrophage-driven hyperinflammation (29), reducing neutrophil extracellular trap formation (34), and clearing pathogens via B-1a cell-derived soluble mediators like IL-10 and natural IgM (31). Considering these findings, it remains uncertain whether the protective effects of B-1a cells can be fully recapitulated by TGF-β alone, and these mediators may act synergistically. Moreover, the long-term effects –such as the survival of B-1a cells, the maintenance of their TGF-β producing capacity, and potential side effects like fibrosis or chronic inflammation— have not been assessed. Future studies using TGF-β–neutralizing antibodies or TGF-β–deficient B-1a cells, investigating the involvement of other B-1a cell products, as well as long-term follow-up experiments, will be important for elucidating the mechanisms and therapeutic potential of B-1a cells.

In summary, our study demonstrates that B-1a cells mitigate acute radiation-induced intestinal injury in mice, with local TGF-β production potentially contributing to this protective effect. These findings elucidate the important roles of B-1a cells and TGF-β in the molecular mechanisms of intestinal regeneration and provide a foundational basis for future strategies aimed at intervention in radiation-induced intestinal injury.

## Data Availability

The original contributions presented in the study are included in the article/**Supplementary Material**. Further inquiries can be directed to the corresponding authors.
