# Supplementary material for: B-1a cells mitigate radiation injury by protecting intestinal barrier integrity
Source: Front Immunol. 2026 Feb 4;17:1761007. doi: 10.3389/fimmu.2026.1761007 (PMC12913145; doi:10.3389/fimmu.2026.1761007)
Supplement: Supplementary file 1 [file DataSheet1.pdf]

Supplemental Figure 1

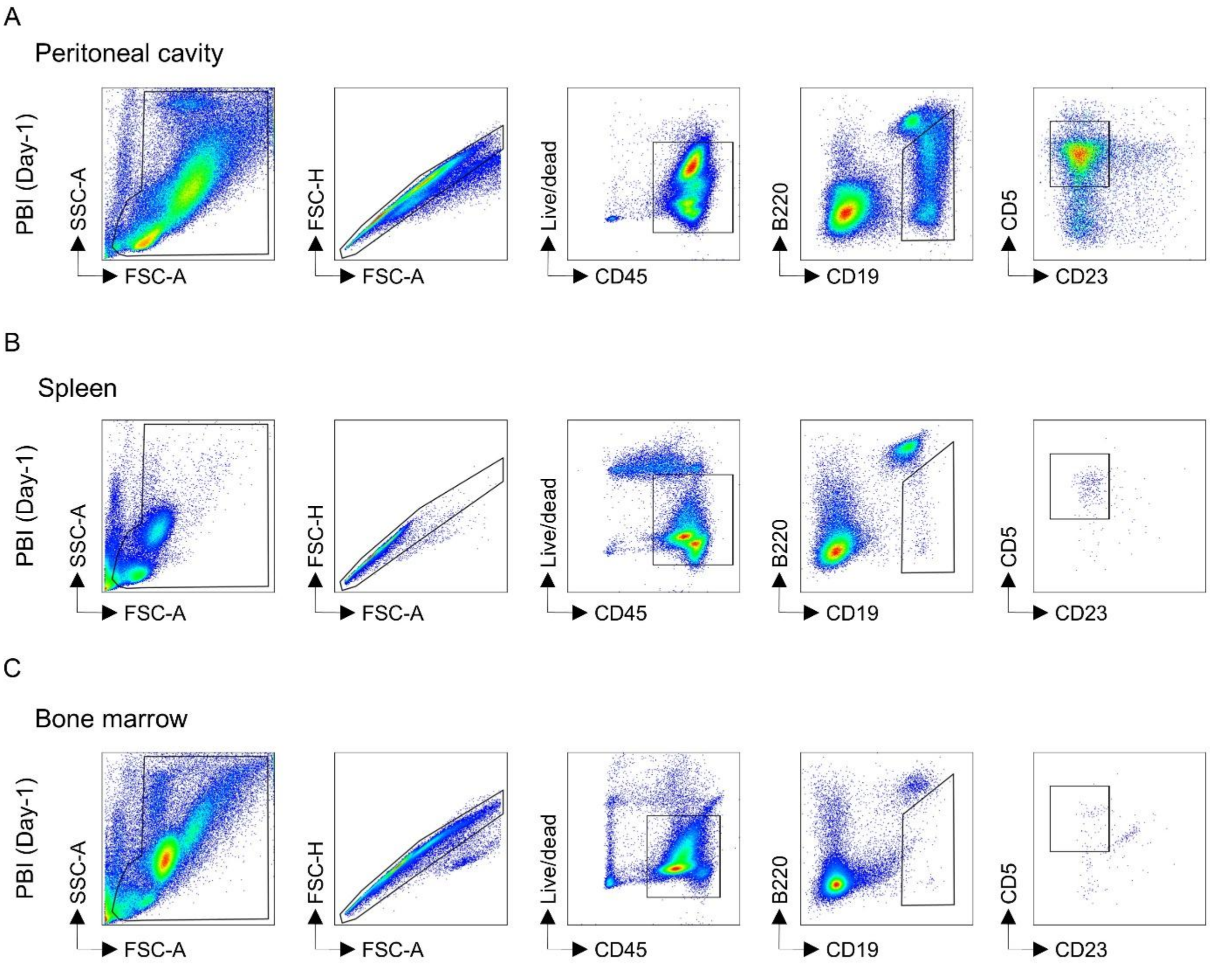

Supplemental Figure 1. Partial body irradiation reduces the number of B-1a cells in the peritoneal cavity, spleen, and bone marrow on day-1. Mice were irradiated with 12-Gy partial body irradiation (PBI) on day-0. Cells from peritoneal cavity (PerC), spleen, and bone marrow were collected on day-1. Representative gating strategy of flow cytometry for B-1a cells ($CD45^{+}CD19^{+}B220^{lo/-}CD23^{-}CD5^{+}$) from **(A)** PerC, **(B)** spleen, and **(C)** bone marrow.

Supplemental Figure 2

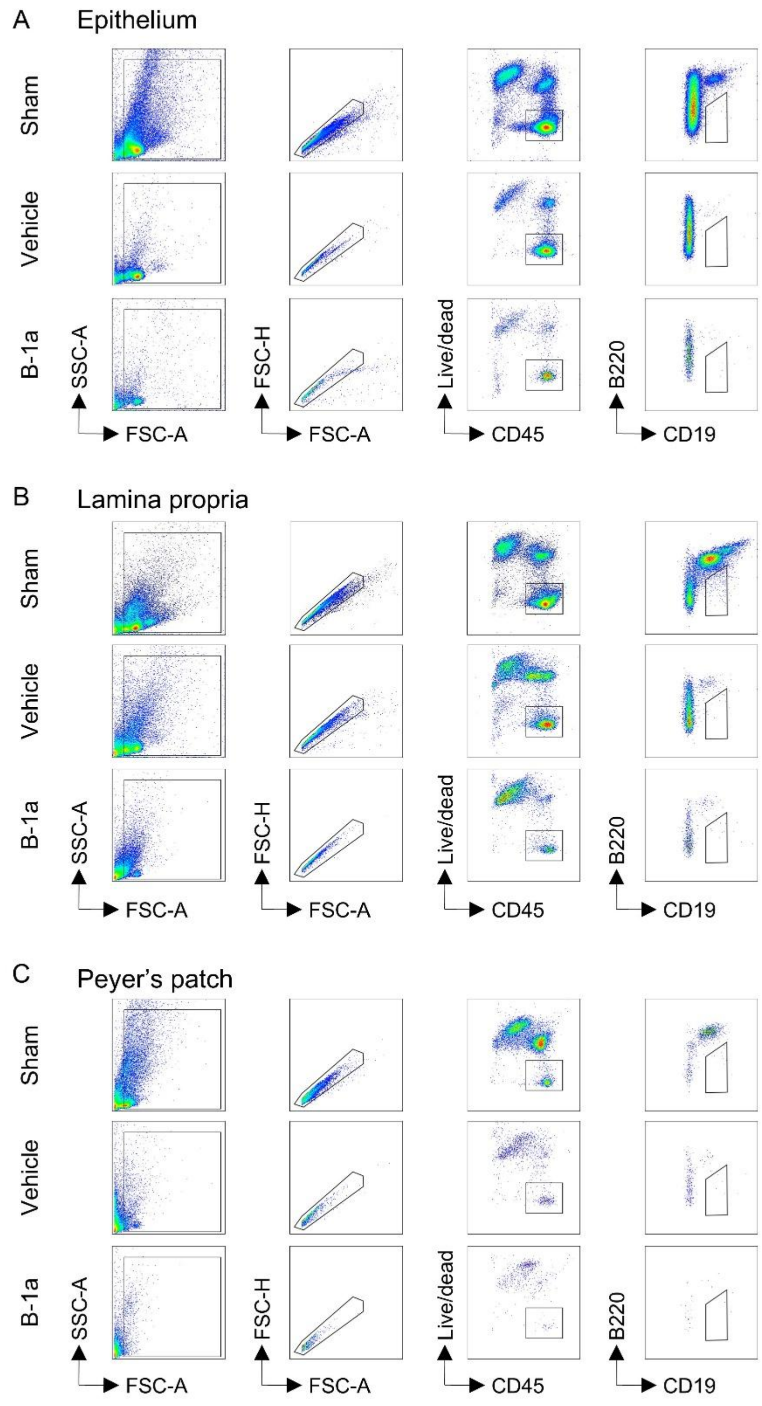

Supplemental Figure 2. B-1 cells are rarely detected in the intestinal epithelium, lamina propria, and Peyer's patches by flow cytometry. Mice were randomly assigned into three groups: sham, vehicle-treated partial body irradiation (PBI), and B-1a cell-treated PBI groups. Mice in vehicle- and B-1a cell-treated PBI groups received 12-Gy PBI on day-0. Mice in B-1a

cell-treated PBI group were administered an intraperitoneal injection of 5×10^5 B-1a cells on day-1, while vehicle group mice received PBS. Small intestines were harvested on day-5. Representative gating strategy of flow cytometry for B-1 cells ($CD45^+CD19^+B220^{lo/-}$) from **(A)** epithelium, **(B)** lamina propria, and **(C)** Peyer's patch.
